# Supplementary figures and images for: A375 melanoma-derived lactate controls A375 melanoma phenotypes by inducing macrophage M2 polarization via TCA cycle and TGF-β signaling
Source: PeerJ. 2025 Feb 21;13:e18887. doi: 10.7717/peerj.18887 (PMC11849511; doi:10.7717/peerj.18887)

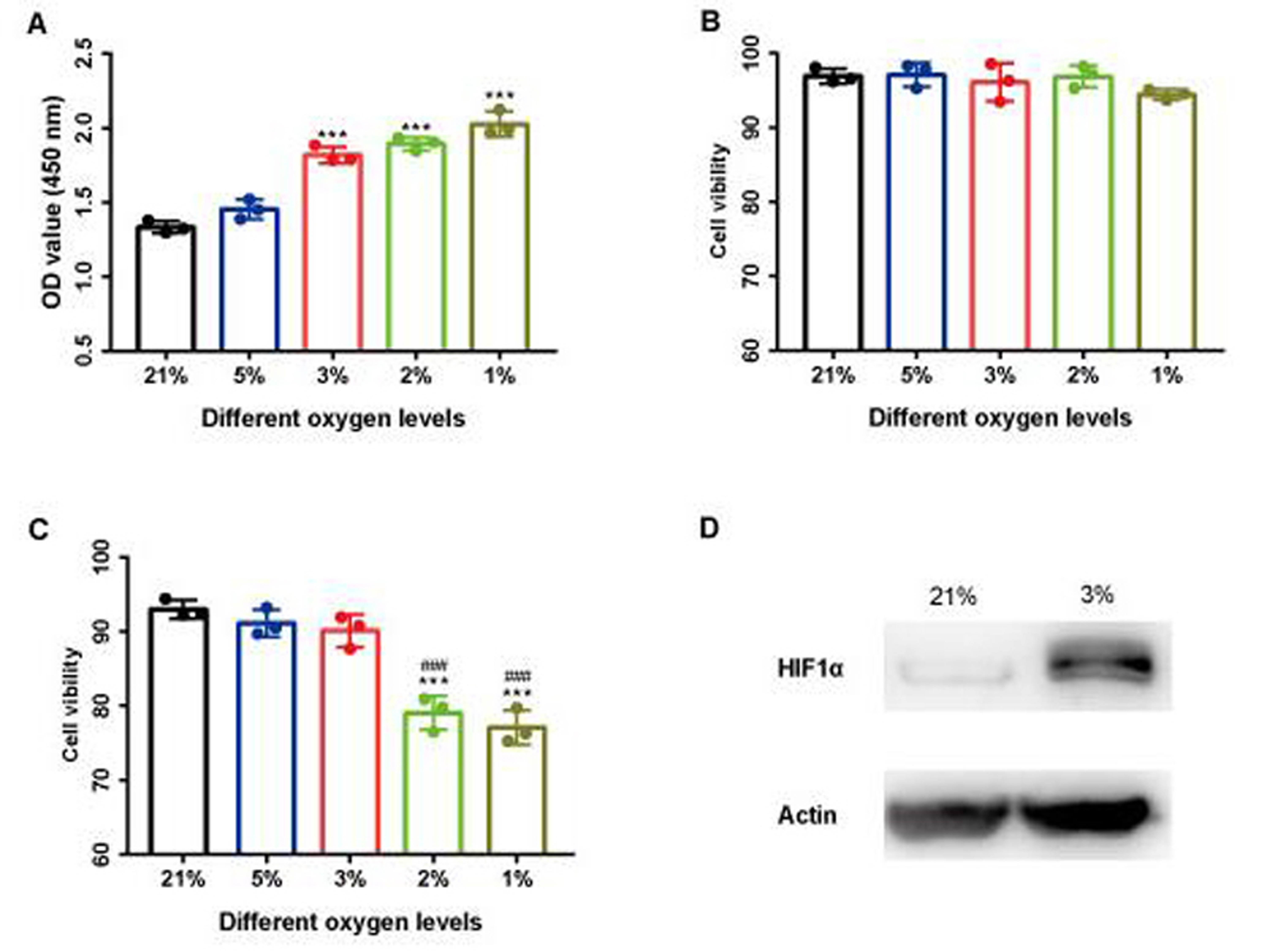

Supplement: Supplemental Information 1 — (A) A375 proliferation under different hypoxic O2 levels. (B-C) A375 cell viability and PBMCs cell viability under hypoxic O2 levels. (D) Western Blot of HIF1α in PBMCs under 3% O2. ***P<0.001 versus 21%, ###P<0.001 verse 3% n=3. [file peerj-13-18887-s001.jpg]

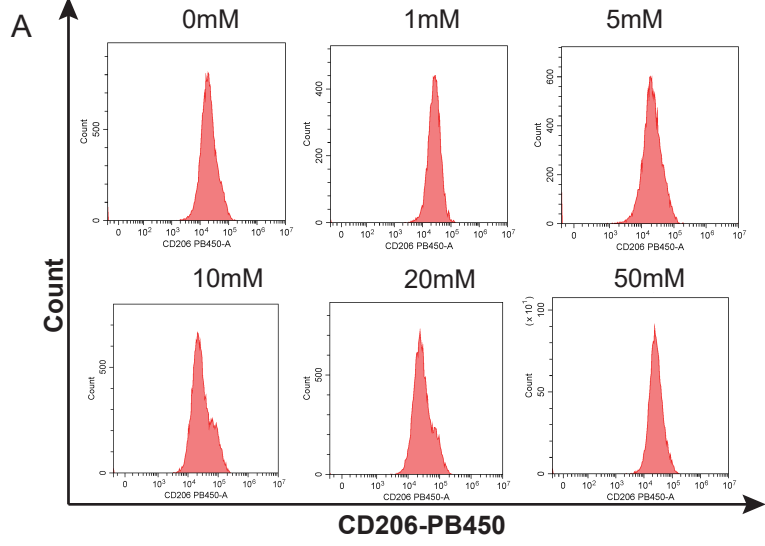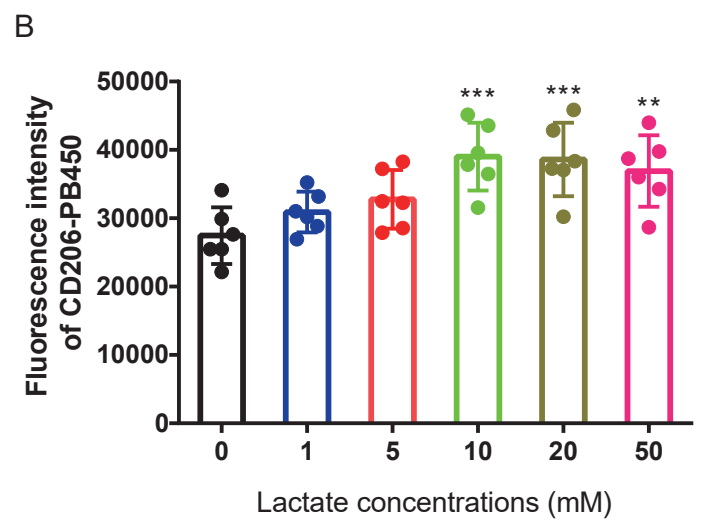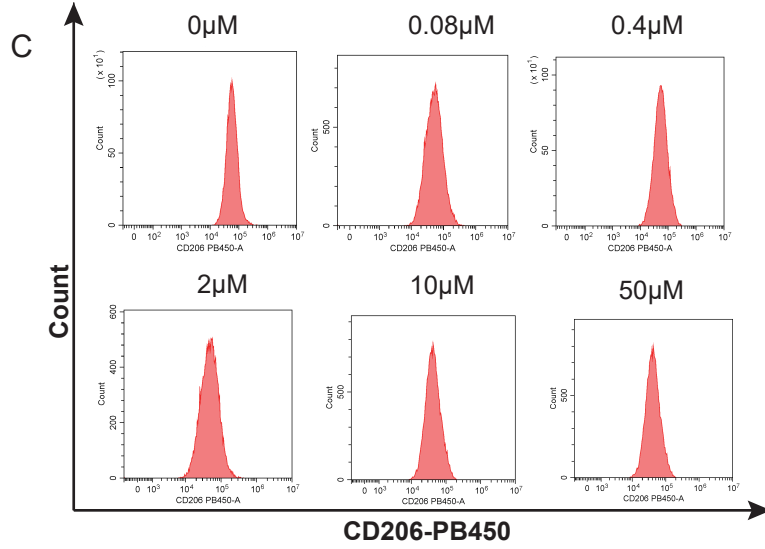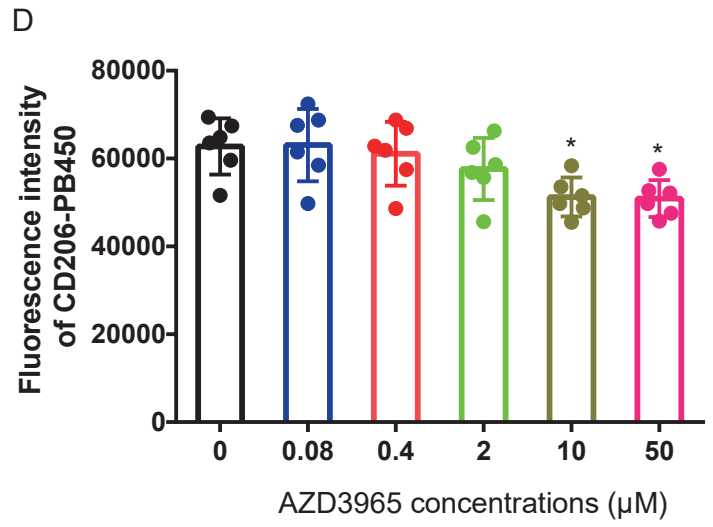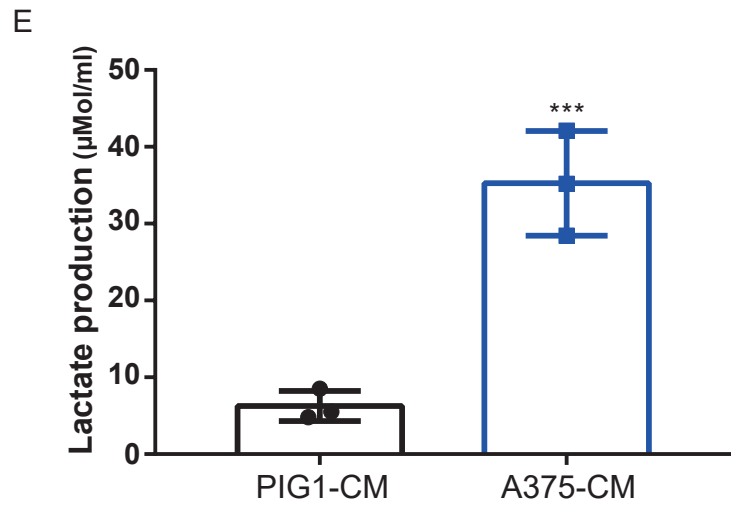

Supplement: Supplemental Information 2 — (A-B) The impact of lactate on CD206 expression in PBMCs. (C-D) The influence of AZD3965 on CD206 expression in PBMCs. (E) Lactate levels in PIG1 and A375 conditioned media. *P< 0.05, **P<0.01, ***P<0.001 versus control, n=3. [file peerj-13-18887-s002.pdf]

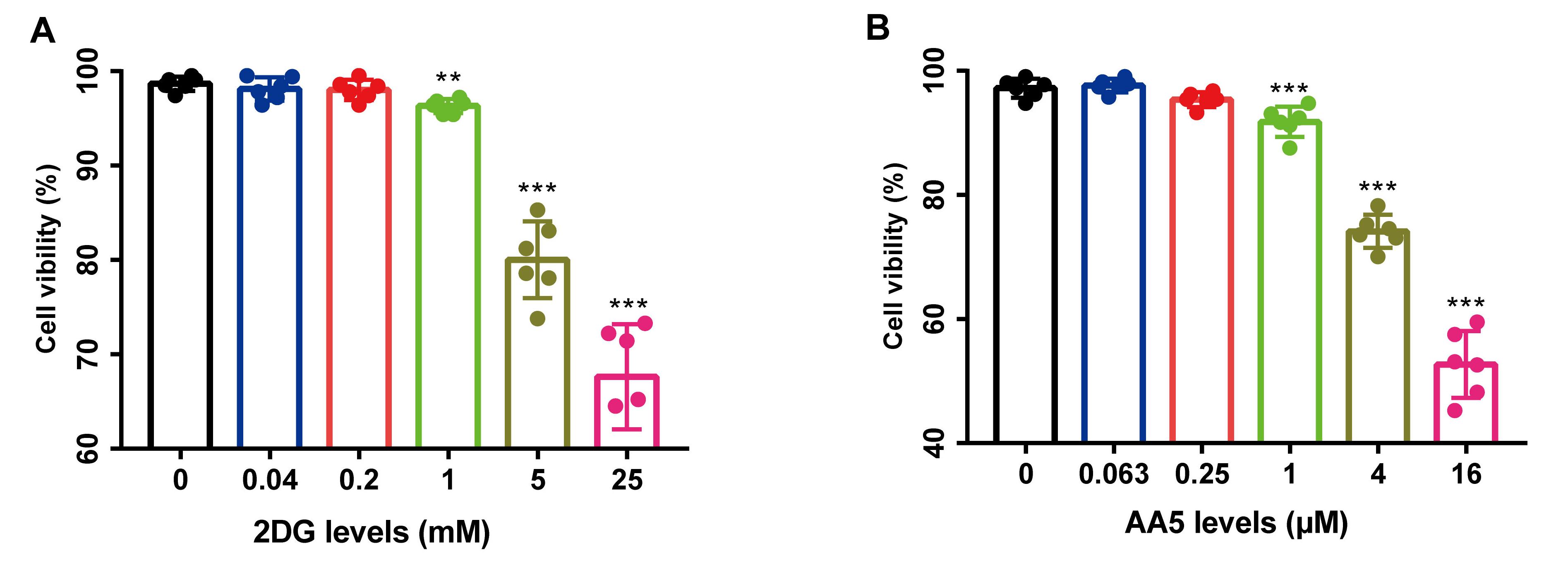

Supplement: Supplemental Information 3 — (A-B) 2DG and AA5 levels were determined via PBMC viability. **P<0.01, ***P<0.001 versus 0, n=3. [file peerj-13-18887-s003.jpg]
